# Virtual Reality Head-Mounted Display (HMD) and Preoperative Patient-Specific Simulation: Impact on Decision-Making in Pediatric Urology: Preliminary Data

**DOI:** 10.3390/diagnostics14151647

**Published:** 2024-07-30

**Authors:** Giulia Lanfranchi, Sara Costanzo, Giorgio Giuseppe Orlando Selvaggio, Cristina Gallotta, Paolo Milani, Francesco Rizzetto, Alessia Musitelli, Maurizio Vertemati, Tommaso Santaniello, Alessandro Campari, Irene Paraboschi, Anna Camporesi, Michela Marinaro, Valeria Calcaterra, Ugo Maria Pierucci, Gloria Pelizzo

**Affiliations:** 1 Department of Pediatric Surgery, Children’s Hospital “Vittore Buzzi”, 20154 Milan, Italy; giulia.lanfranchi@gmail.com (G.L.); sara.costanzo@asst-fbf-sacco.it (S.C.); giorgio.selvaggio@asst-fbf-sacco.it (G.G.O.S.); alessia.musitelli@unimi.it (A.M.); michelamarinaro09@gmail.com (M.M.); ugomariapierucci@icloud.com (U.M.P.); 2 Department of Biomedical and Clinical Sciences “L Sacco”, University of Milano, 20157 Milan, Italy; cristina.gallotta@unimi.it (C.G.); maurizio.vertemati@unimi.it (M.V.); irene.paraboschi01@gmail.com (I.P.); 3 CIMaINa (Interdisciplinary Centre for Nanostructured Materials and Interfaces), University of Milano, 20133 Milan, Italy; paolo.milani@unimi.it (P.M.); tommaso.santaniello@unimi.it (T.S.); 4 Department of Physics “Aldo Pontremoli”, University of Milano, 20133 Milan, Italy; 5 Department of Radiology, ASST Grande Ospedale Metropolitano Niguarda, 20162 Milan, Italy; francesco.rizzetto@unimi.it; 6 Postgraduate School of Diagnostic and Interventional Radiology, University of Milano, 20122 Milan, Italy; 7 Pediatric Radiology and Neuroradiology Unit, “Vittore Buzzi” Children’s Hospital, 20154 Milan, Italy; alessandro.campari@asst-fbs-sacco.it; 8 Pediatric Anesthesia and Intensive Care Unit, “Vittore Buzzi“ Children’s Hospital, 20154 Milan, Italy; anna.camporesi@asst-fbf-sacco.it; 9 Pediatrics and Adolescentology Unit, Department of Internal Medicine, University of Pavia, 27100 Pavia, Italy; valeria.calcaterra@asst-fbf-sacco.it; 10 Pediatric Department, “Vittore Buzzi” Children’s Hospital, 20154 Milan, Italy

**Keywords:** virtual reality, pediatric urology, image-guided surgery, patient-specific, simulation, pediatric oncology

## Abstract

Aim of the Study: To assess how virtual reality (VR) patient-specific simulations can support decision-making processes and improve care in pediatric urology, ultimately improving patient outcomes. Patients and Methods: Children diagnosed with urological conditions necessitating complex procedures were retrospectively reviewed and enrolled in the study. Patient-specific VR simulations were developed with medical imaging specialists and VR technology experts. Routine CT images were utilized to create a VR environment using advanced software platforms. The accuracy and fidelity of the VR simulations was validated through a multi-step process. This involved comparing the virtual anatomical models to the original medical imaging data and conducting feedback sessions with pediatric urology experts to assess VR simulations’ realism and clinical relevance. Results: A total of six pediatric patients were reviewed. The median age of the participants was 5.5 years (IQR: 3.5–8.5 years), with an equal distribution of males and females across both groups. A minimally invasive laparoscopic approach was performed for adrenal lesions (*n* = 3), Wilms’ tumor (*n* = 1), bilateral nephroblastomatosis (*n* = 1), and abdominal trauma in complex vascular and renal malformation (ptotic and hypoplastic kidney) (*n* = 1). Key benefits included enhanced visualization of the segmental arteries and the deep vascularization of the kidney and adrenal glands in all cases. The high depth perception and precision in the orientation of the arteries and veins to the parenchyma changed the intraoperative decision-making process in five patients. Preoperative VR patient-specific simulation did not offer accuracy in studying the pelvic and calyceal anatomy. Conclusions: VR patient-specific simulations represent an empowering tool in pediatric urology. By leveraging the immersive capabilities of VR technology, preoperative planning and intraoperative navigation can greatly impact surgical decision-making. As we continue to advance in medical simulation, VR holds promise in educational programs to include even surgical treatment of more complex urogenital malformations.

## 1. Introduction

Since the onset of the 21st century, the elaboration of magnetic resonance imaging (MRI) and computed tomography (CT) scans into three-dimensional (3D) images has proven invaluable in supporting surgical strategies [1,2].

In recent years, the emerging prominence of virtual reality (VR) in healthcare has enabled surgeons to optimize efficiency and mitigate surgical risks in the operating room [3,4]. VR image-guided surgery has already been integrated into adult clinical practice as a preoperative tool for urological procedures, particularly for addressing adrenal tumors, kidney cancers, and kidney calculi in adult patients [5,6,7]. In the field of urology, VR provides preoperative 3D reconstruction, offering spatial localization and relationships with adjacent anatomical structures, optimal anatomical views, and precise evaluations to facilitate more effective surgical planning [5,6,7].

Given the diverse array of complex diseases affecting children, including congenital urinary tract malformations and kidney and adrenal tumors, pediatric urology demands tailored surgical approaches that prioritize tissue preservation and respect a child’s growth. In particular, the success of minimally invasive approaches in children heavily relies on the surgeon’s comprehension of kidney anatomy and the vascular system.

Moreover, specialized training for pediatric patients, spanning from preterm infants to adolescents, is imperative to grasp and master the intricacies of vascular and urinary tract anatomy, particularly in cases of complex congenital malformations.

Despite the benefits provided by the application of VR navigation systems in adult urology, currently, there are only a few studies examining the application of VR in the field of pediatric urology [8]. Therefore, the study aims to investigate the effectiveness and utility of using patient-specific VR simulations as a supplementary tool in pediatric urology to support minimally invasive urologic procedures in children and decision-making processes.

## 2. Patients and Methods

### 2.1. Patients

The research included pediatric patients diagnosed with urological conditions possibly necessitating surgery and submitted to surgery for renal and adrenal lesions at the Surgical Department of “Vittore Buzzi” Children’s Hospital between January 2021 and December 2023. Patient selection was based on specific criteria including age, medical history, and procedural requirements.

Data collected retrospectively encompassed demographics, preoperative clinical information, instrumental data, intervention details, and postoperative outcomes. CT scans, routinely conducted as part of the diagnostic workup before surgery, were available for all patients. An analysis of the anatomic details from the 3D VR imaging was conducted for each case.

The preoperative setup included evaluation of the critical anatomy, the relationship among vessels and parenchyma, and the correlation between the CT scan and VR Head-Mounted Display (HMD). Navigating the malformation was used to clarify the anatomy preoperatively and the surgical steps to plan vascular dissection and ligation with the aim of renal tissue sparing. Records were postoperatively compared with the intraoperative findings to assess the correlation between 3D reconstruction and VR findings and the influence of preoperative 3D-VR navigation on surgical decision-making.

An experienced pediatric surgeon performed the operations while surgical assistance was provided by a resident physician; the minimally invasive surgery procedure was carried out under general anesthesia.

The study was performed according to the principles outlined in the Declaration of Helsinki, as revised in 2008. Informed written consent was obtained from the parents or legal guardians after having received all the information about the study.

### 2.2. CT Image Processing and 3D VR Scene Building

The 3D VR models for all patients were generated utilizing routine, preoperative, low-dose CT images obtained from a 64-slice scanner (Revolution Evo, GE Healthcare, Chicago, IL, USA). These images were acquired during the venous phase, post intravenous administration of iodinated contrast agent, and were reconstructed with a slice thickness of 0.6 mm on the axial plane. Subsequently, the CT images were retrieved from the institutional PACS (Picture Archiving and Communication System), anonymized, and saved in DICOM format. The exported files were then imported into 3D Slicer v.5.2.1 (www.slicer.org), a freely available and open-source software package for image analysis and advanced visualization. Utilizing pre-built automatic and semi-automatic tools within the software, relevant anatomical structures were segmented, with manual corrections applied as needed under the supervision of a radiologist. These segmentations were utilized to generate 3D surface models, facilitating zooming and viewing from multiple perspectives.

An in-house developed plugin for 3D Slicer was employed to load the 3D models into an HMD via a Universal Serial Bus (USB) connection. The Oculus Quest v.1 (META Inc., Menlo Park, CA, USA), an all-in-one HMD equipped with an OLED display featuring a resolution of 1440–1600 pixels per eye and a refresh rate of 72 Hz, was utilized for this purpose. A previously developed application was adopted to create a dedicated VR environment tailored for visualizing the VR scene and interacting with the 3D models [9,10]. This application enabled motion, rotation, zooming, and adjustment of transparency levels of the 3D VR models through a wireless controller.

### 2.3. Preoperative Evaluation and Patient-Specific Simulation

Preoperative surgical planning underwent rigorous analysis to evaluate the patient’s anatomy and to propose the optimal surgical approach for each case. Surgeons used the HMD to review the 3D models within the VR environment, enabling navigation through the reconstruction and the selective display or concealing of specific anatomical structures during the viewing session. Additionally, certain structures were rendered transparent to facilitate the visualization of embedded structures, which proved essential in effectively examining, for example, a mass within the kidney. The vascular anatomy was meticulously scrutinized to prevent vessel damage, mitigate the risk of bleeding, and ensure optimal tissue preservation. Each clinical case underwent thorough discussion by a multidisciplinary team consisting of pediatric surgeons, radiologists, nephrologists, and anesthesiologists. This collaborative effort aimed to define a detailed anatomical view, ascertain surgery’s advantages and potential risks, and ultimately ensure comprehensive patient care. VR HMD preoperative evaluation of the kidney vessels and pyelic and ureteral variants allowed for identification and easy dissection close to the renal hilum. Images were oriented in the same position as the patient’s on the table to mimic the laparoscopic surgical setup. This preoperative study also allowed a more precise intraoperative trocar positioning for the safest dissection of the lesion.

### 2.4. Intraoperative Support

In the operating room, a dedicated VR workstation was readily accessible to ensure that the surgeons reviewed the preoperative navigation recorded within the VR HMD.

## 3. Results

### 3.1. Patients’ Data

The study comprised six children, with an equal distribution of three females and three males. The cohort included individuals with diverse conditions: three with adrenal masses (two on the right side, one on the left side), one with bilateral nephroblastomatosis, one with Wilms tumor, and one with a complex kidney malformation. Table 1 presents an overview of the baseline characteristics of the included patients.

Two patients (i.e., those with bilateral nephroblastomatosis and those with a unilateral ptotic and hypodysplastic kidney) were considered eligible for an observation-only approach due to the disease findings and patients’ anatomy and symptoms.

Among the remaining four patients, all underwent laparoscopic surgery via a transperitoneal approach. Each patient was positioned according to the preoperative evaluation of the patient-specific simulation.

A trans-umbilical access, a 10 mm, 30-degree laparoscope port for the camera was established, and pneumoperitoneum was achieved using 10 mmHg pressure. Two 5 mm trocars were, respectively, positioned in the epigastrium and ipsilateral hypochondrium. In two cases with lesions on the right side, an additional 3 mm extra port was necessary for liver retraction.

Comprehensive details of both pre- and intraoperative assessments are provided in Table 1, Table 2 and Table 3. The results include any disparities observed between the standard and 3D VR imaging, alterations in the surgical plan influenced by the 3D VR images, and the correlation between the 3D VR imaging and the intraoperative findings.

### 3.2. Adrenal Lesions

**Figure 1:**
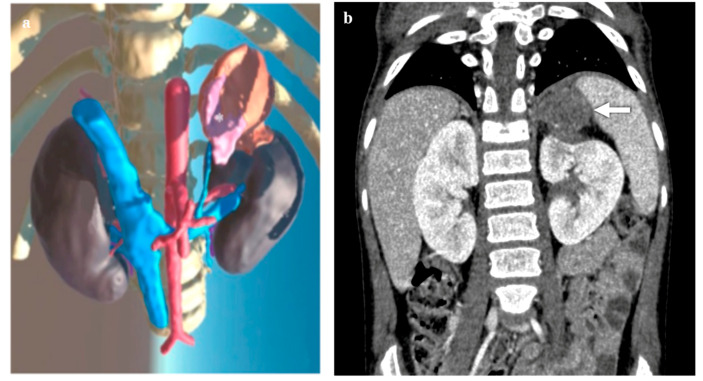
Virtual reality (VR) (**a**) and coronal computed tomography (CT) (**b**) images of a 4-year-old girl with left adrenal neuroblastoma (3.5 × 2.5 × 4 cm in diameter) (* in **a**; → in **b**) in contact with the upper third of the left kidney and the spleen; images show the adrenal vein and the impressions of the adrenal mass on surrounding organs.

**Figure 2:**
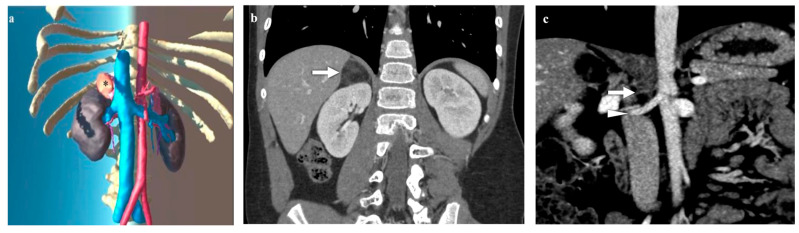
Virtual reality (VR) (**a**) and coronal computed tomography (CT) (**b**,**c**) images of a 7-year-old boy with a right adrenal myelolipoma (* in **a**; → in **b**) measuring 38 × 25 × 25 mm (AP × LL × CC), with heterogeneous density and fatty components. Images depict vascular details: bilateral accessory renal arteries, which on the right course antero-superiorly to the main renal artery heading towards the upper pole, while on the left run postero-superiorly parallel to the main renal artery.

Three patients presented with unilateral adrenal masses subsequently confirmed as neuroblastic tumors in two cases (*n* = 1 neuroblastoma, *n* = 1 ganglioneuroblastoma) and a myelolipoma in the remaining case (Figure 1 and Figure 2). In each case, gross total resections were accomplished, all through laparoscopic interventions. No intraoperative complications were recorded.

The detailed insights provided by VR imaging notably enhanced several technical aspects. Firstly, the 3D visualization of the arterial blood supply anatomy offered a superior view, compared to conventional CT scan studies. Particularly, the ability to navigate in VR through the roots of arterial supply, which can comprise the superior suprarenal artery branching from the inferior phrenic artery, the middle suprarenal artery branching from the abdominal aorta, and the inferior suprarenal artery branching from the renal artery, guided surgeons during the initial phases of dissection. Furthermore, VR provided an enhanced understanding of the relationship between the masses and the adrenal vein. Specifically, in cases of right adrenal masses, where the adrenal vein directly drains into the postero-lateral wall of the inferior vena cava, VR facilitated better visualization than the CT scan study. In such instances, liver retraction and incision of the right triangular ligament aided surgeons in exactly visualizing the adrenal vein drainage into the vena cava. The surgical control of adrenal vein drainage proved to be the most difficult step, and preoperative virtual reality evaluation of the anatomy supported the intraoperative decision-making, which enabled the operation to be started with control of adrenal venous drainage, enabling easy prevention of bleeding.

### 3.3. Kidney Lesions and Congenital Anomalies

**Figure 3:**
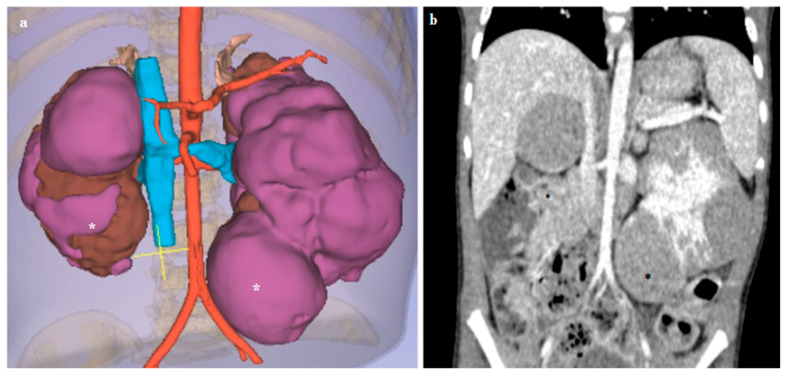
Virtual reality (VR) (**a**) and preoperative computed tomography (CT) (**b**) images of a 3-year-old girl with bilateral nephroblastomatosis (*). Both kidneys are in place, enlarged in size (right kidney 8 cm, left kidney 9 cm), with the parenchymal structure altered due to the presence of multiple solid lesions, the largest of which are located, respectively, at the upper pole of the right kidney, rounded in appearance, approximately 4 cm in diameter, and at the lower pole of the left kidney, approximately 4.5 cm in diameter. The lesions all have a nearly rounded morphology, and defined margins, albeit without a clear capsular structure.

In the case of the patient with bilateral nephroblastomatosis, analyzing the CT scan images revealed intricate details such as multiple arterial blood supplies, the precise location and extension of the masses, and their relationship with adjacent arteries and the excretory system. This analysis suggested the potential for performing a partial nephrectomy. However, the VR navigation system highlighted no issues for a nephron-sparing surgery and a high probability of performing a nephrectomy (Figure 3). Following the latest recommendations for managing patients with bilateral kidney lesions, the patient underwent neoadjuvant chemotherapy until nephron-sparing surgery could be considered feasible [11,12,13,14].

**Figure 4:**
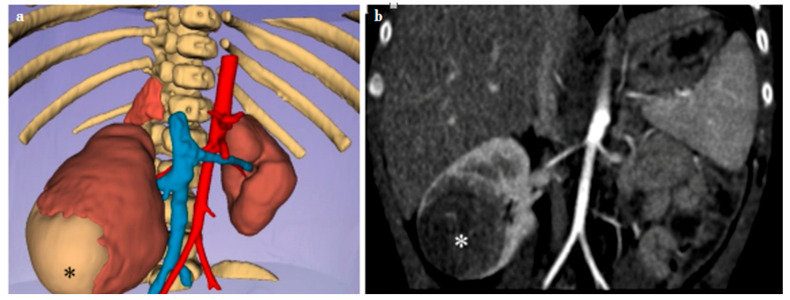
Virtual reality (VR) (**a**) and preoperative computed tomography (CT) (**b**) images of a 3-month-old boy with a right-side Wilms’ tumor (*). The figures show a solid lesion in the middle-lower third of the right kidney (30 × 38 × 30 mm; LL × AP × CC), heterogeneously hypodense compared to the normal renal parenchyma, which posterior-inferior-laterally reaches the renal profile and causes rotation of the renal axis, with posteriorized pelvis. The mass reaches the hilar region. A recognizable single renal artery is present bilaterally. Two right renal venous branches are recognizable, up to near the drainage into the inferior vena cava, the cranial one with a larger caliber. The left kidney is normal.

In the 3-month-old boy with a unilateral Wilms’ tumor involving the middle-lower third of the right kidney (30 × 38 × 30 mm; LL × AP × CC), a laparoscopically assisted radical nephrectomy was performed. The lesion reached the renal profile postero-inferolaterally, was swollen, and caused rotation of the renal axis, with posteriorization of the pelvis and reached the hilar region. A single kidney artery was recognizable bilaterally, while two right kidney venous branches, up to near the drainage into the vena cava, were identified. The cranial one had a larger caliber (Figure 4). Intraoperative decision-making was supported by preoperative navigation and, contrary to what was evidenced from the CT scan, showed the impossibility of performing a heminephrectomy. Overall, the VR offered a valuable platform for studying kidney anatomy in the context of Wilms’ tumor, providing immersive visualization, enhanced spatial awareness, real-time manipulation, simulation of surgical procedures, patient-specific modeling, and opportunities for education and training.

**Figure 5:**
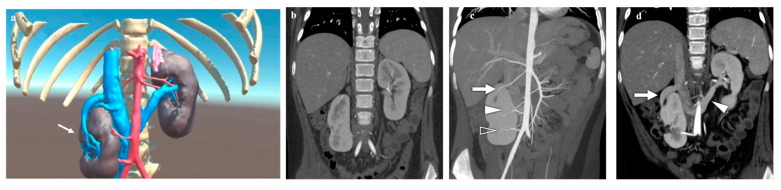
Virtual reality (VR) (**a**) and coronal computed tomography (CT) (**b**–**d**) images of a 10-year-old girl with a symptomatic right ptotic dysmorphic kidney. The figures show a ptotic right kidney, located inferior-medially and rotated, with normal parenchymal thickness. No urinary tract dilatation is noted. Three right renal arteries are present (→): one originates from the proximal aorta, one from the distal aorta, and one from the proximal common iliac artery; on the left, two contiguous renal arteries are depicted, and the left renal vein runs posteriorly to the aorta.

In the patient with a unilateral ptotic and hypoplastic kidney (Figure 5), the VR navigation systems allowed us to study in detail the patient-specific kidney anatomy, which showed an incomplete rotation, with the hilum facing anteriorly and the ureter positioned laterally. VR navigation assessed the correlation between the 3D reconstruction and VR findings on surgical decision-making; due to extremely complex malformation, a conservative approach was chosen and, fortunately, the outcome of abdominal trauma was uneventful.

## 4. Discussion

The adoption of VR technology for planning minimally invasive urological procedures has shown significant growth among adult patients, emerging as a valuable resource for comprehending the patient’s anatomy [15,16,17,18,19]. The literature extensively describes the application of VR in kidney oncologic surgery, highlighting notable changes in surgical strategies and outcomes [5,6,7,20]. Initial clinical investigations involving adults with kidney cancer have demonstrated the promising utility of VR and intracorporeal navigation in enhancing surgical precision during minimally invasive radical or partial nephrectomies. In particular, 3D imaging techniques are beneficial in preserving kidney function and parenchyma [17,21]. VR offers a 3D, immersive environment that allows surgeons to explore and interact with the patient’s anatomy before performing minimally invasive procedures. This technology provides a detailed and accurate representation of the patient’s anatomy, enabling surgeons to plan surgeries more effectively and reduce the risk of complications. One of the key advantages of using VR in urology is its ability to enhance preoperative planning. By visualizing the patient’s anatomy in a virtual environment, surgeons can better understand the spatial relationships between structures and identify potential challenges before entering the operating room. This can lead to more precise surgical plans and improved patient outcomes [8].

Nevertheless, in pediatric patients, there is limited experience in VR application in surgical oncology and there is still no international consensus regarding the utilization of minimally invasive surgery and nephron-sparing surgeries for the management of adrenal and kidney tumors [22,23]. This is mainly attributed to the absence of randomized clinical trials and the restricted sample sizes in retrospective studies [24,25].

Nephron-sparing surgical procedures are especially crucial in scenarios involving congenital malformations (e.g., symptomatic duplex kidney) and in cases of bilateral tumors or solitary kidneys. Bilateral Wilms’ tumor poses notable challenges, as the treatment objectives strive to attain sufficient oncological radicality for definitive treatment, while concurrently conserving renal function and minimizing long-term morbidity via nephron-sparing surgery. In such situations, VR provides an invaluable platform for exploring kidney anatomy, offering immersive visualization, improved spatial understanding, real-time interaction, surgical procedure simulation, personalized patient models, and educational and training opportunities.

Our study focused on investigating the application of VR in pediatric urology among a cohort of pediatric patients with various diagnoses. We have observed, anecdotally, that surgeries utilizing VR technology tend to have shorter operation times and reduced blood loss, highlighting the practical advantages of VR in surgical practice. Furthermore, we encountered no complications or need for conversion to open surgery.

Additionally, our study underscored alterations in the surgical plan for cases with kidney diseases. Enhanced visualization of vascular anatomy, including intricate details, such as small tumor vessels, and comprehension of the relationship between masses and hilar vessels, were notable benefits of VR reconstruction. Preoperative planning was enhanced by VR reconstruction, allowing enhanced visualization of potential variants and the morphology of the vascular collecting system, thereby aiding in avoiding potential surgical challenges. Moreover, significant concordance was observed between the 3D model and intraoperative anatomy. As a result, one of the most significant advantages of navigation is the comprehensive evaluation of the vascular tree, enabling surgeons to select the optimal route to reach the target while minimizing the risk of vascular injuries.

Within the field of surgical education, there has been limited urology-specific research exploring the utilization of VR [8]. Urologists in training may encounter challenges in attaining proficiency due to inherent technical complexities and limited case exposure. Consequently, there is an increasing interest in exploring alternatives to the traditional surgical apprenticeship model, leading to the emergence of various simulation programs employing virtual trainers [26]. VR simulation-based training holds promise in supplementing conventional training for trainees with limited or no prior laparoscopic urological experience. The era of surgical training has evolved beyond the paradigm of “See one, do one, teach one,” towards multi-level theoretical and practical, hands-on surgical training to ensure patient safety and treatment effectiveness. Improved surgical education methods are particularly crucial in pediatric urology, where the complexity of patient diseases and the physical size of patients pose challenges to existing surgical technologies and skills.

While VR shows promise in studying specific complex cases and planning surgical strategies, our study has limitations, including a small sample size and manual segmentation of the 3D model. Future developments should focus on the automation of software to streamline imaging processing. Additionally, prospective randomized studies with larger sample sizes are warranted to assess the true benefits of VR technology for complex pediatric urologic diseases. Despite these limitations, our study represents a significant step in understanding the real impact of VR in defining and planning the optimal surgical strategy and in comparing perioperative and intraoperative findings in pediatric urology.

## 5. Conclusions

This study underscores the potential of patient-specific VR simulations as a valuable complement to decision-making and care provision in pediatric urological procedures. Integrating VR simulations into preoperative planning offers a more nuanced understanding of patient-specific anatomy and pathology, enabling tailored treatment strategies in most pediatric urological cases. Additionally, it boosts healthcare provider’s confidence and proficiency by providing a realistic rehearsal environment. This, in turn, has the potential to mitigate intraoperative risks, ultimately enhancing patient outcomes. While our study presents promising results, future research should include long-term follow-up studies to assess the sustainability of observed improvements, even in the case of complex pediatric urogenital malformations.

VR should also be considered as a surgical education tool in training curricula for non-surgical techniques, decision-making, and communication in pediatric urology.

## Figures and Tables

**Table 1:** Overview of the characteristics of the patients included in the study.

Patient	Gender	Age	Lesion	Side
Case n. 1	Female	4 years	Neuroblastoma	Left
Case n. 2	Male	7 years	Adrenal myelolipoma	Right
Case n. 3	Male	9 years	Ganglioneuroblastoma	Right
Case n. 4	Female	3 years	Nephroblastomatosis	Bilateral
Case n. 5	Male	3 months	Wilms’ tumor	Right
Case n. 6	Female	10 years	Ptotic and hypodysplastic kidney	Right

**Table 2:** Advantages and disadvantages of preoperative assessment using computed tomography (CT) scans or virtual reality (VR) navigation systems.

Anatomical Structures	Computed Tomography (CT) Scans	Virtual Reality (VR) Navigation Systems
Adrenal gland vasculature	A high cognitive load for the visualization of small blood vessels and finer vascular structures directed to the adrenal gland. Visibility of vascular structures may be limited by the enhancement of adjacent organs or tissues, leading to potential misinterpretation of CT findings.	Highly immersive, three-dimensional environment and detailed visualization of the complex vasculature of the adrenal gland. Easy preoperative visualization of blood vessel localization, course, and branching patterns. Limitations in differentiating between soft tissues and surrounding adrenal tissue.
Kidney hilum	High cognitive load on small anatomical structures and visualization of finer details within the kidney hilum, potentially leading to an incomplete or inaccurate depiction of vascular anatomy. Insufficient depth perception.	VR technology offers a deeply immersive and three-dimensional representation, enabling detailed observation of the intricate anatomical structures within the kidney hilum. Surgeons can explore the hilum from diverse perspectives, gaining comprehensive insights into its spatial relationships and vascular configuration.
Kidney parenchyma	Inadequate clarity of segmentation and orientation on conventional CT planes. Inadequate soft tissue contrast can impede the accurate identification of renal tissue.	Preoperative planning enhances the precision and accuracy of nephron-sparing surgery by enabling surgeons to precisely localize renal lesions, demarcate tumor boundaries, and strategize tailored resection plans related to vascular supply.

**Table 3:** Virtual reality (VR) navigation systems in preoperative urologic assessment: impact on surgical decision-making.

Advantages	Debatable Values
Improved precision in identifying vascular anatomy and detecting variants.	Variable accuracy in identifying the vascular supply of the calyces and renal pelvis.
High precision in the orientation of arteries and veins to the parenchyma.	
High accuracy in depth perception.	

## Data Availability

Data reported in this study are available upon request from the corresponding author.
